# Branched‐chain amino acids and sleep: a population‐derived study of Australian children aged 11–12 years and their parents

**DOI:** 10.1111/jsr.13855

**Published:** 2023-02-23

**Authors:** Lisa Matricciani, Dorothea Dumuid, Catherine Paquet, Kurt Lushington, Tim Olds

**Affiliations:** ^1^ Clinical & Health Sciences University of South Australia Adelaide Australia; ^2^ Alliance for Research in Exercise, Nutrition and Activity (ARENA) University of South Australia Adelaide Australia; ^3^ Allied Health and Human Performance (AHHP) University of South Australia Adelaide Australia; ^4^ Murdoch Children's Research Institute Parkville Victoria Australia; ^5^ Faculté des Sciences Administratives Université Laval Quebec City Quebec Canada; ^6^ Centre Nutrition, santé et société (NUTRISS), INAF Université Laval Quebec City Quebec Canada; ^7^ Centre de Recherche, Centre Hospitalier Universitaire de Québec – Université Laval Quebec City Quebec Canada; ^8^ Centre for Behaviour‐Brain‐Body: Justice and Society Unit University of South Australia Adelaide South Australia Australia

**Keywords:** branched‐chain amino acids, micronutrients, sleep

## Abstract

Micronutrients, particularly amino acids, are thought to play an important role in sleep regulation and maintenance. While tryptophan is a known predictor of sleep, less is known about branched‐chain amino acids (BCAAs), which compete with tryptophan for transport across the blood–brain barrier. The aim of this study was to determine the association between BCAAs and actigraphy‐derived sleep duration, timing and efficiency, and self‐reported trouble sleeping. This study examined data on children and adults collected as part of the Child Health CheckPoint study. Linear mixed models, adjusted for geographic clustering, were used to determine the association between BCAAs and sleep characteristics. Complete‐case analysis was conducted for 741 children aged 11–12 years old (51% females) and 941parents (87% mothers). While BCAAs were significantly associated with children's sleep duration, timing and self‐reported trouble sleeping, no associations were observed in adults, in fully adjusted models. In children, higher levels of BCAAs are associated with shorter sleep duration, delayed sleep timing, and more frequent reports of trouble sleeping.

## 1 INTRODUCTION

Diet has long been recognised as important for sleep. Various foods, such as cow's milk, cherries, and kiwi fruit have been shown to promote sleep, while others, such as caffeine and alcohol, have been shown to disrupt sleep (Frank et al., 2017; St‐Onge et al., 2016). The effects of food on sleep are thought to be explained by the action of macronutrients (such as carbohydrates, proteins, and fats) and micronutrients (such as vitamins, minerals, amino acids, and lipids). Of the micronutrients, amino acids are known to play an important role.

Tryptophan is an essential amino acid that has been associated with increased subjective sleepiness, reduced latency to sleep onset, and fewer night‐time awakenings (Silber & Schmitt, 2010; Sutanto et al., 2022). Tryptophan is thought to cross the blood–brain barrier where it is involved in the synthesis of serotonin – a precursor of the sleep‐promoting hormone melatonin (Binks et al., 2020). Although tryptophan promotes sleep, it competes with branched‐chain amino acids (BCAAs) at the crossing of the blood–brain barrier (Binks et al., 2020; Humer et al., 2020). BCAAs are therefore thought to promote wakefulness by reducing tryptophan transport through the blood–brain barrier and hence the synthesis of serotonin (Humer et al., 2020). Since both tryptophan and BCAAs are essential amino acids and cannot be produced *de novo*, a diet rich in tryptophan may only improve sleep when BCAAs are low. While competition with tryptophan transport is one mechanism in which BCAAs may influence sleep, other mechanisms may also exist. For instance, BCAAs are involved in the synthesis of *de novo* glutamate and GABA, neurotransmitters also known to influence sleep (Falup‐Pecurariu et al., 2021; Holeček, 2018).

Few studies have examined the role of BCAAs in sleep regulation and maintenance. Studies on BCAA supplements typically focus on muscle mass development, fatigue and performance (Fedewa et al., 2019; Fouré & Bendahan, 2017). While sleep is often not the focus of these studies, BCAA supplements are thought to reduce central fatigue, delay sleep onset, and impair sleep quality, especially if taken later in the day (Hormoznejad et al., 2019; Ordóñez et al., 2017; Portier et al., 2008). In line with these studies, Xiao et al. (2017) in their survey of 277 Chinese adults, reported BCAAs were significantly associated with delayed sleep timing. Consistent findings were also reported by Gordon‐Dseagu et al. (2019) in a study of 106 adults. While biological pathways suggest elevated BCAA levels in healthy individuals have a negative impact on sleep, clinical studies (paradoxically) suggest BCAA supplements are a viable treatment option for sleep disturbances in patients with traumatic brain injury (Elliott et al., 2018; Elliott et al., 2022; Lim et al., 2013). In these cases, BCAAs are provided as supplements for *de novo* synthesis of glutamate and GABA, which are known to promote sleep, but are deficient after a traumatic brain injury.

To date, population‐based studies that have examined the association between BCAAs and sleep in adults are limited and under‐explored in children. Moreover, available studies have relied on self‐reported measures of sleep duration and timing with their attendant biases (Gordon‐Dseagu et al., 2019; Xiao et al., 2017). In line with contemporary conceptualisations of sleep and methods of assessing sleep, it is important to consider actigraphy‐derived measures of sleep alongside self‐report measures of sleep quality (Buysse, 2014; Matricciani et al., 2018). Given the preliminary evidence and physiological basis for a potential relationship between BCAAs and sleep, as well as the availability and use of supplements containing BCAAs, there is a need to extend our understanding of the relationship between BCAAs and sleep. Therefore, the aim of this study was to test whether higher BCAAs levels (isoleucine, leucine, and valine) were associated with sleep (actigraphic and self‐report measures) in a large population‐derived sample of Australian children and their parents.

## 2 METHODS

This study examines data collected as part of the Child Health CheckPoint study, a one‐off cross‐sectional study nested between Waves 6 and 7 of the Longitudinal Study of Australian Children (LSAC). The CheckPoint study was conducted between February 2015 and March 2016 and involved a comprehensive physical health and biomarker assessments of children aged 11–12 years, and one of their parents. Further details of the CheckPoint study has been provided elsewhere (Edwards, 2014; Sanson & Johnstone, 2004).

### 2.1 Ethics and consent

The CheckPoint study protocol was approved by The Royal Children's Hospital Melbourne Human Research Ethics Committee (33225D) and Australian Institute of Family Studies Ethics Committee (14–26). The attending parent/caregiver provided written informed consent for themselves and their child to participate in the study.

### 2.2 Sleep

Sleep was assessed in terms of actigraphy‐derived sleep characteristics and self‐reported trouble sleeping.

#### 2.2.1 Actigraphy‐derived sleep

GENEActiv monitors (Activinsights), fitted to the participant's non‐dominant wrist, were used to assess sleep. Participants were asked to wear the monitor continuously for 8 consecutive days. Raw acceleration data, collapsed into 60 s epochs, were processed using *Cobra* software (Fraysse et al., 2019) to derive three objective characteristics of sleep examined in this study:

- Sleep period (the difference between sleep onset and offset),
- Sleep midpoint (the midpoint between sleep onset and offset),
- Sleep efficiency (the percent of minutes scored as sleep between onset and offset).

Participants were included for analysis if they had at least 4 nights of sleep data recorded, had an average sleep time > 200 min, and at least 1 weekend night (Friday or Saturday night) of sleep data. These criteria were predetermined by the Child Health CheckPoint team to reflect habitual sleep (Fraysse et al., 2019). Further details of sleep data processing in this study have been reported elsewhere (Fraysse et al., 2019; Matricciani et al., 2019). All actigraphy‐derived sleep variables were computed for each individual day and then averaged using a 5:2 weighting for weeknight (Sunday–Thursday) and weekend (Friday–Saturday).

#### 2.2.2 Trouble sleeping

Trouble sleeping was assessed via self‐report. Participants were asked to report how often they had trouble sleeping over the past month, using a 5‐point Likert scale (never, almost never, sometimes, often, always).

#### 2.2.3 Covariates

Covariates selected for this study included socio‐economic position, maturity (age of adults and pubertal stage of children), sex, and body mass index (BMI). These variables were selected as covariates as they have been associated with both sleep and metabolomics profiles, particularly BCAAs (Dollman et al., 2007; Felden et al., 2015; Jarrin et al., 2014; Ohayon et al., 2004; Olds et al., 2010). For children, pubertal stage rather than age was selected as a covariate as the children examined in this study were within a narrow age range (11–12 years) and since complex changes in amino acid metabolism have been observed during puberty (Cominetti et al., 2020). Puberty was assessed using the Puberty Development Scale, a validated self‐report questionnaire that consists of five Likert scale questions (Chan et al., 2010; Petersen et al., 1988). A higher Puberty Development Scale score represents more advanced pubertal development. For parents, age was calculated from date of birth and expressed as years. Socio‐economic position (SEP) was determined using a standardised scale derived from the LSAC dataset, which reflects household income, education, and occupation (Baker et al., 2017; Blakemore et al., 2006). Higher SEP scores reflect higher socio‐economic position. Adult alcohol consumption was determined from Wave 6 LSAC questionnaire data, using a continuous self‐report measure of average daily alcohol consumption, calculated from the midpoint of categories related to frequency and quantity of alcohol consumption. BMI (kg/m^2^) z‐score and waist‐to‐hip ratio (used in sensitivity analysis) was determined using anthropometry measures. For children, BMI *z*‐score were calculated using the Centers for Disease Control CDC reference dataset (Clifford et al., 2019).

### 2.3 Branched‐chain amino acids

All three branched‐chain amino acids (BCAAs) (leucine, isoleucine, and valine) were examined in this study. BCAA concentrations were derived using semi‐fasted venous blood samples, taken from consenting children and adults participating in the CheckPoint study. Appropriately trained researchers or phlebotomists collected venous blood samples within the assessment centres. Blood collection included four Becton Dickinson (BD) Vacutainer tubes collected in the order of: 2.7 mL EDTA, 9 mL EDTA, 9 mL serum, 7.5 mL lithium heparin, which were then processed at an on‐site processing laboratory (Ellul et al., 2019). Samples were spun at room temperature for 10 min at 550× g relative centrifugal force before 0.5 mL aliquots were taken of plasma, serum, buffy coat lymphocytes, whole blood and/or a blood clot (Ellul et al., 2019). Samples were then immediately frozen at −80°C for batch analysis at the Melbourne Children's Bioresource Center (Murdoch Children's Research Institute). Metabolomic profiling was done using the Nightingale NMR metabolomics platform (Helsinki, Finland) using the 2016‐version quantification algorithm (Ellul et al., 2019). A high‐throughput experimental setup was used to simultaneously measure a range of metabolites, including (but not limited to) routine lipids, lipoproteins, fatty acids, glycolysis‐related metabolites, and amino acids, measured from 0.35 mL serum (Ellul et al., 2019). This process generated 228 serum metabolites including nine amino acids (Ellul et al., 2019). Further detail of blood collection, storage, and metabolomics processing has been reported elsewhere (Ellul et al., 2019).

### 2.4 Statistical analysis

Data management and analyses were undertaken in R (version 4.1.0). The association between each of the three BCAAs (predictor) and the different sleep characteristics (outcome) were assessed using linear mixed models, with the postal code entered as a random intercept to account for the geographic clustering. Mixed models are a suitable technique as they allow for non‐independent correlation structures and account for between‐ and within‐group variance (Harrison et al., 2018). Geographical clustering was an important consideration as data were collected in specific towns and suburbs across Australia where differences might exist in health‐related outcomes. To account for potential differences related to geographic location, mixed models were used. Postal code was selected as it is a large spatial unit that correspond to towns and suburbs. The *performance* package in R was used to confirm linear mixed model assumptions were met (linearity, homogeneity of variance, collinearity, normality of residuals, normality of random effects, posterior predictive check, influential observations) (Lüdecke et al., 2021). Complete case analysis was undertaken in R, version 4.1.0, using the lme4 package (Bates et al., 2015). Two models were undertaken for each of the four sleep characteristics. The first model was adjusted for age (of parents)/puberty stage (of children), sex, SEP, average alcohol consumption (of parents), and fasting time. The second model adjusted sociodemographic characteristics in addition to BMI z‐score. Analyses were undertaken for children and adults separately. Sensitivity analysis was performed with waist‐to‐hip ratio instead of BMI z‐score to verify the robustness of results.

## 3 RESULTS

### 3.1 Participant characteristics

Of the 1874 children and their parents who took part in the CheckPoint Study, complete data were available for 741 children and 941 adults. Participants in this study were relatively healthy and few had diabetes (2% of adults and 0.1% of children). Demographic details of the included participants are presented in Table 1. As shown, the mean age of adults was 38.8 years, with the majority being female (87%). The mean age of children was 11.9 (SD 0.4) years, with an approximately equal number of boys and girls. Participants had a higher SEP z‐score than the population‐based sample of LSAC B cohort participants (mean = 0.32, SD = 0.90 vs. mean = 0.00, SD = 1.00) (Blakemore et al., 2009). Self‐report trouble sleeping was poorly correlated with actigraphy‐derived sleep parameters in children (*r* = −0.05 to 0.07) and adults (*r* = −0.07 to 0.07).

**TABLE 1:** Descriptive statistics of participants included for analysis

Children	
*N*	741
Age	11.9 (0.4)
Sex (*n* (%) females)	379 (51.1)
Born in Australia (*n* (%))	735 (99.2)
SEP	0.32 (0.90)
Puberty development scale	2.09 (0.57)
BMI	19.05 (3.31)
Waist‐to‐hip ratio	0.82 (0.05)
Sleep midpoint (24 h:min)	02:38 (45)
Sleep period (min)	568 (46)
Sleep efficiency (%)	84 (6)
Trouble sleeping (*n*)	
Never	226 (30.5)
Almost never	252 (34.0)
Sometimes	160 (21.6)
Often	73 (9.9)
Always	30 (4.0)
Isoleucine (mmol/L)	0.05 (0.02)
Leucine (mmol/L)	0.07 (0.02)
Valine (mmol/L)	0.16 (0.04)
Median fasting time (h:min) [median, IQR]	4.1 (3.4–4.8)
Adults	
*N*	941
Born in Australia (*n* (%))	765 (81.3)
Age (years)	38.8(4.5)
Sex (*n* (%) females)	817 (86.8)
SEP (mean (SD))	0.27 (0.97)
BMI	27.4 (5.8)
Waist‐to‐hip ratio	0.95 (0.09)
Smokes cigarettes (*n* (%))	10.2 (7.6)
Daily alcohol consumption (standard drinks)	0.55 (0.79)
Sleep midpoint (24 h:min)	02:48 (49)
Sleep period (min)	498 (55)
Sleep efficiency (%)	86 (6)
Trouble sleeping (*n*)	
Never	86 (9.1)
Almost never	362 (38.5)
Sometimes	353 (37.5)
Often	113 (12.0)
Always	27 (2.9)
Isoleucine (mmol/L)	0.05 (0.02)
Leucine (mmol/L)	0.07 (0.02)
Valine (mmol/L)	0.16 (0.04)
Fasting time (h:min) [median, IQR]	2.9 (2.2–3.9)

*Note*: Unless otherwise reported mean (SD) values are reported.

Abbreviation: BMI, body mass index; SEP, socioeconomic position.

### 3.2 Branched‐chain amino acids and sleep of children

BCAAs were significantly associated with sleep parameters in children. As presented in Table 2, leucine was significantly associated with all sleep parameters, while isoleucine was significantly associated with all sleep characteristics except sleep efficiency (Model 1). Valine was significantly associated with sleep duration (β = −0.11; CI = −0.18 to −0.03; *p* = 0.004) and timing (β = 0.08; CI = 0.01 to 0.15; *p* = 0.031), even after adjusting for BMI z‐score (duration: β = −0.09; CI = −0.16 to −0.02; *p* = 0.015; timing: β = 0.08; CI = 0.00 to 0.15; *p* = 0.043) (Model 1). After adjusting for BMI z‐score, isoleucine and leucine were no longer significant predictors of sleep duration (Model 2). Observed associations were mostly in the expected direction, higher levels of BCAAs were associated with shorter sleep duration, later sleep timing, and more troubled sleep. While significant associations were observed, effect sizes were small. For instance, one standard deviation increase in leucine was associated with a − 0.07 standard deviation decrease in sleep duration and 0.09 standard deviation increase in sleep timing, reflecting a decrease of 3.2 min/day in sleep duration and a 4.1 min/day delay in sleep timing. Associations observed for sleep efficiency were in the direction opposite to expected (higher BCAAs were associated with higher sleep efficiency), however, effect sizes were very small (0.36%). Sensitivity analysis was performed with waist‐to‐hip ratio instead of BMI z‐score to verify the robustness of results. Results relating to the relationship between BCAAs and sleep were not affected and are presented in Appendix A.

**TABLE 2:** Linear mixed models for the association between BCAAs and sleep characteristics in children

	Sleep duration						Sleep timing						Sleep efficiency							Trouble sleeping				
	Model 1			Model 2			Model 1			Model 2			Model 1			Model 2			Model 1			Model 2		
	β	SE	*p*‐Value	β	SE	*p*‐Value	β	SE	*p*‐Value	β	SE	*p*‐Value	β	SE	*p*‐Value	β	SE	*p*‐Value	β	SE	*p*‐Value	β	SE	*p*‐Value
Intercept	0.00	0.037	**<0.001**	0.00	0.037	**<0.001**	0.00	0.039	**<0.001**	0.00	0.039	**<0.001**	0.00	0.036	**<0.001**	0.00	0.036	**<0.001**	0.00	0.036	**<0.001**	0.00	0.036	**<0.001**
Sex	0.08	0.038	**0.044**	0.07	0.038	0.075	0.03	0.038	0.408	0.03	0.038	0.388	0.12	0.038	**0.002**	0.13	0.038	**0.001**	−0.02	0.038	0.63	−0.02	0.038	0.552
SEP	−0.01	0.037	0.818	−0.01	0.037	0.77	−0.09	0.037	**0.021**	−0.09	0.037	**0.022**	−0.01	0.037	0.898	0.00	0.037	0.948	−0.08	0.036	**0.028**	−0.08	0.036	**0.026**
Puberty	−0.08	0.039	**0.029**	−0.06	0.039	0.133	0.08	0.038	**0.043**	0.07	0.039	0.062	−0.02	0.038	0.575	−0.04	0.039	0.263	0.16	0.038	**<0.001**	0.17	0.039	**<0.001**
Isoleucine	−0.09	0.037	**0.022**	−0.07	0.038	0.085	0.10	0.037	**0.006**	0.10	0.038	**0.010**	0.05	0.037	0.205	0.03	0.038	0.448	0.09	0.037	**0.017**	0.10	0.037	**0.009**
Fasting time	−0.05	0.037	0.191	−0.04	0.037	0.252	0.02	0.037	0.592	0.02	0.037	0.613	0.08	0.037	**0.042**	0.07	0.037	0.059	−0.04	0.037	0.246	−0.04	0.037	0.279
zBMI				−0.11	0.038	**0.006**				0.02	0.038	0.626				0.10	0.038	**0.010**				−0.05	0.038	0.184
Intercept	0.00	0.037	**<0.001**	0.00	0.037	**<0.001**	0.00	0.039	**<0.001**	0.00	0.039	**<0.001**	0.00	0.036	**<0.001**	0.00	0.036	**<0.001**	0.00	0.036	**<0.001**	0.00	0.036	**<0.001**
Sex	0.07	0.038	0.056	0.07	0.038	0.088	0.04	0.038	0.351	0.04	0.038	0.335	0.13	0.038	**0.001**	0.13	0.038	**0.001**	−0.02	0.038	0.697	−0.02	0.038	0.623
SEP	−0.01	0.037	0.831	−0.01	0.037	0.782	−0.09	0.037	**0.019**	−0.09	0.037	**0.020**	0.00	0.037	0.915	0.00	0.036	0.963	−0.08	0.036	**0.025**	−0.08	0.036	**0.023**
Puberty	−0.08	0.039	**0.029**	−0.06	0.039	0.129	0.08	0.038	**0.043**	0.07	0.039	0.062	−0.02	0.038	0.586	−0.04	0.039	0.286	0.16	0.038	**<0.001**	0.17	0.039	**<0.001**
Leucine	−0.09	0.037	**0.013**	−0.07	0.038	0.063	0.09	0.037	**0.012**	0.09	0.038	**0.019**	0.08	0.037	**0.042**	0.06	0.038	0.139	0.08	0.036	**0.039**	0.09	0.037	**0.021**
Fasting time	−0.05	0.037	0.21	−0.04	0.037	0.269	0.01	0.037	0.693	0.01	0.037	0.713	0.08	0.037	**0.035**	0.07	0.037	**0.047**	−0.05	0.036	0.192	−0.05	0.036	0.218
zBMI				−0.10	0.038	**0.008**				0.02	0.038	0.634				0.09	0.038	**0.018**				−0.05	0.038	0.186
Intercept	0.00	0.037	**<0.001**	0.00	0.037	**<0.001**	0.00	0.039	**<0.001**	0.00	0.039	**<0.001**	0.00	0.036	**<0.001**	0.00	0.036	**<0.001**	0.00	0.036	**<0.001**	0.00	0.036	**<0.001**
Sex	0.07	0.038	0.084	0.06	0.038	0.122	0.04	0.038	0.301	0.04	0.038	0.283	0.13	0.038	**0.001**	0.14	0.038	**<0.001**	−0.01	0.038	0.721	−0.02	0.038	0.668
SEP	0.00	0.037	0.96	−0.01	0.037	0.882	−0.09	0.037	**0.013**	−0.09	0.037	**0.014**	−0.01	0.037	0.813	−0.01	0.036	0.887	−0.09	0.036	**0.019**	−0.09	0.036	**0.017**
Puberty	−0.08	0.038	**0.034**	−0.06	0.039	0.141	0.08	0.038	**0.050**	0.07	0.039	0.076	−0.02	0.038	0.553	−0.04	0.039	0.263	0.16	0.038	**<0.001**	0.17	0.039	**<0.001**
Valine	−0.11	0.037	**0.004**	−0.09	0.037	**0.015**	0.08	0.037	**0.031**	0.08	0.037	**0.043**	0.07	0.037	0.051	0.06	0.037	0.131	0.04	0.036	0.230	0.05	0.037	0.172
Fasting time	−0.04	0.037	0.225	−0.04	0.036	0.271	0.01	0.036	0.792	0.01	0.036	0.814	0.07	0.037	**0.042**	0.07	0.037	0.053	−0.05	0.036	0.138	−0.05	0.036	0.150
zBMI				−0.10	0.038	**0.007**				0.03	0.038	0.516				0.09	0.038	**0.014**				−0.04	0.038	0.288

*Note*: Random intercepts used to adjust for clustering by postcode. Bold *p* values indicate significance <0.05.

Abbreviation: zBMI, body mass index z‐score; SE, standard error; SEP, socioeconomic position; β, standardised coefficient.

### 3.3 Branched‐chain amino acids and sleep of adults

As shown in Table 3, BCAAs were not significantly associated with any of the actigraphy‐derived sleep parameters in adults. Isoleucine (β = 0.10; CI = 0.03 to 0.16; *p* = 0.004) and leucine (β = 0.09; CI = 0.02 to 0.15; *p* = 0.011) were significantly associated with self‐reported trouble sleeping (Model 1), even after adjusting for BMI z‐score (isoleucine; β = 0.08; CI = 0.01 to 0.15; *p* = 0.022; leucine; β = 0.07; CI = 0.00 to 0.14; *p* = 0.040) (Model 2). Sensitivity analysis was performed with waist‐to‐hip ratio instead of BMI z‐score to verify the robustness of results. Results relating to the relationship between BCAAs and sleep were not affected and are presented in Appendix B.

**TABLE 3:** Linear mixed models for the association between BCAAs and sleep characteristics in adults

	Sleep duration						Sleep timing						Sleep efficiency							Trouble sleeping				
	Model 1			Model 2			Model 1			Model 2			Model 1			Model 2			Model 1			Model 2		
	β	SE	*p*‐Value	β	SE	*p*‐Value	β	SE	*p*‐Value	β	SE	*p*‐Value	β	SE	*p*‐Value	β	SE	*p*‐Value	β	SE	*p*‐Value	β	SE	*p*‐Value
Intercept	0.00	0.035	**<0.001**	0.00	0.035	**<0.001**	−0.01	0.036	**<0.001**	−0.01	0.036	**<0.001**	0.00	0.034	**<0.001**	0.00	0.033	**<0.001**	0.00	0.032	**0.001**	0.00	0.032	**0.001**
Sex	0.12	0.034	**0.001**	0.12	0.034	**0.001**	−0.01	0.034	0.798	−0.01	0.034	0.796	0.05	0.035	0.13	0.05	0.035	0.127	0.14	0.034	**<0.001**	0.14	0.034	**<0.001**
SEP	−0.07	0.033	**0.032**	−0.07	0.033	**0.028**	−0.07	0.033	**0.048**	−0.06	0.034	0.062	0.09	0.033	**0.010**	0.07	0.034	**0.028**	−0.14	0.032	**<0.001**	−0.13	0.033	**<0.001**
Age	−0.04	0.033	0.182	−0.05	0.033	0.177	0.06	0.034	0.104	0.06	0.034	0.101	−0.01	0.034	0.679	−0.02	0.034	0.63	0.06	0.033	0.091	0.06	0.033	0.080
Isoleucine	0.01	0.034	0.751	0.02	0.035	0.667	0.01	0.034	0.687	0.01	0.035	0.794	−0.03	0.034	0.365	−0.01	0.035	0.741	0.10	0.033	**0.004**	0.08	0.035	**0.022**
Alcohol use	0.06	0.032	0.061	0.06	0.032	0.064	−0.05	0.032	0.096	−0.05	0.033	0.100	0.02	0.033	0.523	0.02	0.033	0.578	0.03	0.032	0.444	0.03	0.032	0.398
Fasting time	0.05	0.032	0.148	0.05	0.032	0.139	−0.03	0.033	0.341	−0.03	0.033	0.323	−0.01	0.033	0.816	0.00	0.033	0.957	0.04	0.032	0.26	0.03	0.032	0.331
zBMI				−0.02	0.034	0.659				0.02	0.035	0.66				−0.07	0.035	0.058				0.06	0.034	0.105
Intercept	0.00	0.035	**<0.001**	0.00	0.035	**<0.001**	−0.01	0.036	**<0.001**	−0.01	0.036	**<0.001**	0.00	0.034	**<0.001**	0.00	0.033	**<0.001**	0.00	0.032	**0.002**	0.00	0.032	**0.001**
Sex	0.11	0.034	**0.001**	0.11	0.034	**0.001**	−0.02	0.035	0.611	−0.02	0.035	0.616	0.06	0.035	0.111	0.06	0.035	0.115	0.14	0.034	**<0.001**	0.14	0.034	**<0.001**
SEP	−0.07	0.033	**0.028**	−0.07	0.033	**0.027**	−0.07	0.033	**0.041**	−0.06	0.034	0.058	0.09	0.033	**0.009**	0.07	0.034	**0.027**	−0.15	0.032	**<0.001**	−0.14	0.033	**<0.001**
Age	−0.05	0.033	0.180	−0.05	0.033	0.178	0.05	0.034	0.105	0.06	0.034	0.101	−0.01	0.034	0.677	−0.02	0.034	0.628	0.06	0.033	0.088	0.06	0.033	0.077
Leucine	−0.01	0.034	0.791	−0.01	0.035	0.846	−0.02	0.034	0.65	−0.02	0.035	0.555	−0.02	0.034	0.554	0.00	0.035	0.907	0.09	0.034	**0.011**	0.07	0.034	**0.040**
Alcohol use	0.06	0.032	0.062	0.06	0.032	0.064	−0.05	0.032	0.095	−0.05	0.033	0.103	0.02	0.033	0.496	0.02	0.033	0.571	0.02	0.032	0.544	0.02	0.032	0.469
Fasting time	0.05	0.032	0.165	0.05	0.032	0.16	−0.03	0.033	0.302	−0.04	0.033	0.279	−0.01	0.033	0.835	0.00	0.033	0.976	0.04	0.032	0.268	0.03	0.032	0.345
zBMI				−0.01	0.034	0.78				0.02	0.034	0.512				−0.07	0.034	**0.046**				0.06	0.034	0.065
Intercept	0.00	0.035	**<0.001**	0.00	0.035	**<0.001**	−0.01	0.036	**<0.001**	−0.01	0.036	**<0.001**	0.00	0.034	**<0.001**	0.00	0.033	**<0.001**	0.00	0.032	**0.001**	0.00	0.032	**0.001**
Sex	0.12	0.034	**0.001**	0.12	0.034	**0.001**	−0.02	0.034	0.503	−0.02	0.034	0.518	0.06	0.034	0.068	0.06	0.034	0.079	0.13	0.034	**<0.001**	0.13	0.034	**<0.001**
SEP	−0.07	0.033	**0.029**	−0.07	0.033	**0.027**	−0.07	0.033	**0.042**	−0.06	0.034	0.065	0.09	0.033	**0.008**	0.07	0.034	**0.028**	−0.15	0.032	**<0.001**	−0.14	0.033	**<0.001**
Age	−0.04	0.033	0.183	−0.05	0.033	0.18	0.05	0.034	0.109	0.05	0.034	0.105	−0.01	0.034	0.686	−0.02	0.034	0.635	0.06	0.033	0.089	0.06	0.033	0.076
Valine	0.01	0.033	0.842	0.01	0.034	0.79	−0.04	0.034	0.251	−0.04	0.034	0.204	0.00	0.034	0.909	0.02	0.034	0.609	0.06	0.033	0.058	0.05	0.034	0.142
Alcohol use	0.06	0.032	0.062	0.06	0.032	0.065	−0.06	0.032	0.088	−0.05	0.032	0.096	0.02	0.033	0.503	0.02	0.033	0.57	0.02	0.032	0.485	0.03	0.032	0.418
Fasting time	0.05	0.032	0.151	0.05	0.032	0.144	−0.04	0.033	0.266	−0.04	0.033	0.242	0.00	0.033	0.891	0.00	0.033	0.964	0.03	0.032	0.289	0.03	0.032	0.376
zBMI				−0.01	0.034	0.706				0.03	0.034	0.445				−0.07	0.034	**0.033**				0.07	0.033	**0.039**

*Note*: Random intercepts used to adjust for clustering by postcode. Bold *p* values indicate significance <0.05.

Abbreviation: zBMI, body mass index z‐score; SE, standard error; SEP, socioeconomic position; β, standardised coefficient.

## 4 DISCUSSION

This study examined the association between BCAAs and different sleep characteristics in a large, population‐derived sample of Australian children and their parents (mostly mothers). This study found that while BCAAs were significantly associated with sleep in children, they were not in adults. Higher levels of BCAAs in children were associated with a shorter sleep duration, later sleep timing, and more frequent reports of trouble sleeping.

This is the first study to examine the association between BCAAs and sleep in terms of a range of actigraphy‐derived sleep parameters and self‐reported trouble sleeping. The current study extends our understanding of the relationship between BCAAs and sleep. All prior studies that we are aware of, rely on self‐reported measures of sleep and examine only two characteristics of sleep (Gordon‐Dseagu et al., 2019; Xiao et al., 2017). Gordon‐Dseagu et al. (2019) in a sample of 106 adults enrolled in the Dietary Approaches to Stop Hypertension (DASH)‐sodium feeding trial, found BCAAs were associated with sleep timing but not sleep duration. Consistent with this study, Xiao and colleagues, in a study of 277 Chinese men, also found BCAAs were associated with sleep timing but not sleep duration (Xiao et al., 2017). The authors of this study noted that most participants were healthy sleepers and that a larger sample size is needed to determine effects on sleep duration. In adults, we observed no association with sleep duration or sleep timing. The former but not the latter findings are consistent with those reported by Xiao et al. (2017) and Gordon‐Dseagu et al. (2019). The failure to find a relationship with sleep timing is difficult to explain, but the present study did include a larger sample with comparable mean (SD) sleep values to those of prior studies. It is possible that adult sleep, particularly that of mothers, is explained by a wide range of external factors, that small effects observed in this study are not detected as being significant in this group.

Although BCAAs were not associated with adult sleep, significant associations were observed for children's sleep duration, timing, and self‐reported trouble sleeping, even after adjusting for sex, puberty stage, SEP, and BMI z‐score. Biological processes related to growth and development (puberty) may explain why different associations are observed for children (Cominetti et al., 2020; Terasawa, 2005). In particular, puberty has been shown to involve changes in amino acid metabolism, with complex patterns specifically observed for BCAAs (Cominetti et al., 2020). Further, while the mechanisms of puberty onset are complex, laboratory‐based studies suggest the GABAergic neuronal system plays an important role, with changes observed in glutamate and gamma‐aminobutyric acid (GABA) concentrations, substrates that BCAAs are known to be involved in the synthesis of (Holeček, 2018; Terasawa, 2005). However, this area of research has not been extensively explored. Dietary patterns and parenting styles associated with socioeconomic position may also, in part, explain findings. Whole foods, such as chicken, eggs, salmon, nuts, and brown rice are rich in BCAAs, but are atypically preferred by children. Higher consumption of BCAAs in children may therefore reflect a more health‐conscious parenting style, which may also include more after‐school activities and stimulating pre‐bed activities (such as homework) that may result in later bedtimes and poorer sleep. Reverse causality may also be possible, however, the cross‐sectional nature of this study precludes our ability to infer causality.

### 4.1 Strength and weaknesses

This study examines the association between BCAAs and sleep in a large, population‐derived sample of Australian children and their parents. Key strengths include the large samples of both children and adults, as well as a comprehensive range of sleep parameters measured by both actigraphy and self‐report. Despite these strengths, there are also a number of limitations that need to be acknowledged. Firstly, care must be taken when generalising findings as included participants were of slightly higher SEP than the general population with a z‐score 0.32 (0.90) vs 0.00 (1.00). Adult participants were all parents, and most (87%) were mothers. Child participants were from a narrow age range (11–12 years). Secondly, since tryptophan was not present in the publicly available CheckPoint dataset used in this study, the relative influence of tryptophan remains unclear. It is also important to note that this study only examined the role of BCAAs. Other large neutral amino acids, such as tyrosine and phenylalanine, may also compete with tryptophan in passing the blood–brain barrier. Thirdly, although analyses were adjusted for fasting time, diet prior to fasting was not measured. Fourthly, this study was a cross‐sectional analysis and cannot imply causality. Lastly, although different dimensions of sleep were examined, actigraphy‐derived sleep parameters were assessed over a relatively short period of a week and single‐item self‐report trouble sleeping has not been validated as a specific dimension of sleep (Matricciani et al., 2022).

### 4.2 Future directions

This study reveals BCAAs are significantly associated with children's sleep. Future interventions that target sleep through dietary modifications may need to consider the role of BCAAs. Diet and BCAA consumption may also be important to consider among people, particularly children experiencing poor sleep. Screening BCAA consumption through dietary questionnaires and/or blood assays may help to identify whether diet is a contributing factor of poor sleep patterns and/or complaints. However, the relationship between BCAAs and sleep has not been examined extensively. Further efforts are needed to understand the possible biological mechanisms that underpin our findings and how biological processes during puberty affect BCAA metabolism and sleep regulation.

## 5 CONCLUSIONS

This study finds BCAAs are significantly associated with children's sleep, albeit the effect sizes are small. Higher levels of BCAAs were associated with shorter sleep duration, delayed sleep timing, and more frequent reports of trouble sleeping among children. No association was observed for adults.

## AUTHOR CONTRIBUTIONS

LM conceived the study, prepared the initial draft, participated in the writing and preparation of the manuscript and the analysis and interpretation of data; DD participated in the writing and preparation of the manuscript and the analysis and interpretation of the data; CP participated in the writing and preparation of the manuscript and the analysis and interpretation of the data; KL participated in the writing and preparation of the manuscript and the analysis and interpretation of the data; TO participated in the writing and preparation of the manuscript and the analysis and interpretation of the data.

## FUNDING INFORMATION

DD is supported by an Australian National Health and Medical Research Council (NHMRC) Early Career Fellowship APP1162166. Maurizio Costabile for reading a draft of this manuscript.

## CONFLICT OF INTEREST

The authors have no conflicts to declare.

## Data Availability

The data that support the findings of this study are openly available to researchers at no cost under licence. Data access requests are co‐ordinated by the National Centre for Longitudinal Data (see https://dataverse.ada.edu.au/dataverse/lsac).

## APPENDIX A Linear mixed models for the association between BCAAs and sleep characteristics in children

**Table:**

	Sleep duration						Sleep timing						Sleep efficiency							Trouble sleeping				
	Model 1			Model 2			Model 1			Model 2			Model 1			Model 2			Model 1			Model 2		
	β	SE	*p*‐Value	β	SE	*p*‐Value	β	SE	*p*‐Value	β	SE	*p*‐Value	β	SE	*p*‐Value	β	SE	*p*‐Value	β	SE	*p*‐Value	β	SE	*p*‐Value
Intercept	0.00	0.037	**<0.001**	0.00	0.037	**<0.001**	0.00	0.039	**<0.001**	0.00	0.039	**<0.001**	0.00	0.036	**<0.001**	0.00	0.036	**<0.001**	0.00	0.036	**<0.001**	0.00	0.036	**0.005**
Sex	0.08	0.038	**0.044**	0.08	0.038	**0.029**	0.03	0.038	0.408	0.03	0.038	0.478	0.12	0.038	**0.002**	0.12	0.038	**0.003**	−0.02	0.038	0.63	−0.02	0.038	0.664
SEP	−0.01	0.037	0.818	−0.02	0.037	0.693	−0.09	0.037	**0.021**	−0.08	0.037	**0.028**	−0.01	0.037	0.898	0.00	0.037	0.968	−0.08	0.036	**0.028**	−0.08	0.036	**0.025**
Puberty	−0.08	0.039	**0.029**	−0.07	0.039	0.076	0.08	0.038	**0.043**	0.07	0.039	0.08	−0.02	0.038	0.575	−0.04	0.039	0.366	0.16	0.038	**<0.001**	0.16	0.039	**<0.001**
Isoleucine	−0.09	0.037	**0.022**	−0.08	0.037	**0.045**	0.10	0.037	**0.006**	0.10	0.037	**0.011**	0.05	0.037	0.205	0.04	0.037	0.315	0.09	0.037	**0.017**	0.09	0.037	**0.015**
Fasting time	−0.05	0.037	0.191	−0.04	0.037	0.258	0.02	0.037	0.592	0.02	0.037	0.675	0.08	0.037	**0.042**	0.07	0.037	0.063	−0.04	0.037	0.246	−0.04	0.037	0.266
Waist:hip				−0.07	0.038	0.055				0.05	0.038	0.215				0.07	0.038	0.063				−0.02	0.038	0.61
Intercept	0.00	0.037	**<0.001**	0.00	0.037	**<0.001**	0.00	0.039	**<0.001**	0.00	0.039	**<0.001**	0.00	0.036	**<0.001**	0.00	0.036	**<0.001**	0.00	0.036	**<0.001**	0.00	0.036	**0.007**
Sex	0.07	0.038	0.056	0.08	0.038	**0.037**	0.04	0.038	0.351	0.03	0.038	0.421	0.13	0.038	**0.001**	0.12	0.038	**0.002**	−0.02	0.038	0.697	−0.01	0.038	0.731
SEP	−0.01	0.037	0.831	−0.01	0.037	0.706	−0.09	0.037	**0.019**	−0.08	0.037	**0.026**	0.00	0.037	0.915	0.00	0.037	0.958	−0.08	0.036	**0.025**	−0.08	0.036	**0.023**
Puberty	−0.08	0.039	**0.029**	−0.07	0.039	0.075	0.08	0.038	**0.043**	0.07	0.039	0.08	−0.02	0.038	0.586	−0.03	0.039	0.386	0.16	0.038	**<0.001**	0.16	0.039	**<0.001**
Leucine	−0.09	0.037	**0.013**	−0.08	0.037	**0.028**	0.09	0.037	**0.012**	0.09	0.037	**0.021**	0.08	0.037	**0.042**	0.07	0.037	0.077	0.08	0.036	**0.039**	0.08	0.037	**0.034**
Fasting time	−0.05	0.037	0.21	−0.04	0.037	0.276	0.01	0.037	0.693	0.01	0.037	0.778	0.08	0.037	**0.035**	0.07	0.037	0.05	−0.05	0.036	0.192	−0.05	0.037	0.208
Waist:hip				−0.07	0.038	0.06				0.05	0.038	0.208				0.07	0.038	0.081				−0.02	0.038	0.632
Intercept	0.00	0.037	**<0.001**	0.00	0.037	**<0.001**	0.00	0.039	**<0.001**	0.00	0.039	**<0.001**	0.00	0.036	**<0.001**	0.00	0.036	**<0.001**	0.00	0.036	**<0.001**	0.00	0.036	**0.006**
Sex	0.07	0.038	0.084	0.07	0.039	0.056	0.04	0.038	0.301	0.03	0.038	0.374	0.13	0.038	**0.001**	0.12	0.039	**0.002**	−0.01	0.038	0.721	−0.01	0.038	0.747
SEP	0.00	0.037	0.96	−0.01	0.037	0.819	−0.09	0.037	**0.013**	−0.09	0.037	**0.019**	−0.01	0.037	0.813	0.00	0.037	0.955	−0.09	0.036	**0.019**	−0.09	0.036	**0.018**
Puberty	−0.08	0.038	**0.034**	−0.07	0.039	0.083	0.08	0.038	**0.05**	0.07	0.039	0.094	−0.02	0.038	0.553	−0.04	0.039	0.36	0.16	0.038	**<0.001**	0.16	0.039	**<0.001**
Valine	−0.11	0.037	**0.004**	−0.10	0.037	**0.008**	0.08	0.037	**0.031**	0.07	0.037	**0.049**	0.07	0.037	0.051	0.06	0.037	0.087	0.04	0.036	0.23	0.05	0.037	0.217
Fasting time	−0.04	0.037	0.225	−0.04	0.037	0.288	0.01	0.036	0.792	0.01	0.037	0.878	0.07	0.037	**0.042**	0.07	0.037	0.059	−0.05	0.036	0.138	−0.05	0.036	0.146
Waist:hip				−0.07	0.038	0.063				0.05	0.038	0.181				0.07	0.038	0.074				−0.01	0.038	0.738

*Note*: Random intercepts used to adjust for clustering by postcode.

Abbreviations: SE, standard error; SEP, socioeconomic position; Waist:hip, waist‐to‐hip ratio; β, standardised coefficient.

## APPENDIX B Linear mixed models for the association between BCAAs and sleep characteristics in adults

**Table:**

	Sleep duration						Sleep timing						Sleep efficiency							Trouble sleeping				
	Model 1			Model 2			Model 1			Model 2			Model 1			Model 2			Model 1			Model 2		
	β	SE	*p*‐Value	β	SE	*p*‐Value	β	SE	*p*‐Value	β	SE	*p*‐Value	β	SE	*p*‐Value	β	SE	*p*‐Value	β	SE	*p*‐Value	β	SE	*p*‐Value
Intercept	0.00	0.035	**<0.001**	0.00	0.035	**<0.001**	−0.01	0.036	**<0.001**	−0.01	0.036	**<0.001**	0.00	0.034	**<0.001**	0.00	0.033	**<0.001**	0.00	0.032	**0.001**	0.00	0.032	0.311
Sex	0.12	0.034	**0.001**	0.12	0.034	**0.001**	−0.01	0.034	0.798	−0.01	0.034	0.774	0.05	0.035	0.13	0.06	0.035	0.109	0.14	0.034	**<0.001**	0.13	0.034	**<0.001**
SEP	−0.07	0.033	**0.032**	−0.06	0.033	0.079	−0.07	0.033	**0.048**	−0.06	0.034	0.064	0.09	0.033	**0.010**	0.07	0.033	**0.027**	−0.14	0.032	**<0.001**	−0.13	0.033	**<0.001**
Age	−0.04	0.033	0.182	−0.04	0.033	0.188	0.06	0.034	0.104	0.06	0.034	0.102	−0.01	0.034	0.679	−0.02	0.033	0.658	0.06	0.033	0.091	0.06	0.033	0.085
Isoleucine	0.01	0.034	0.751	−0.02	0.035	0.595	0.01	0.034	0.687	0.01	0.036	0.881	−0.03	0.034	0.365	−0.01	0.036	0.871	0.10	0.033	**0.004**	0.07	0.035	**0.047**
Alcohol use	0.06	0.032	0.061	0.06	0.032	0.073	−0.05	0.032	0.096	−0.06	0.033	0.091	0.02	0.033	0.523	0.02	0.032	0.473	0.03	0.032	0.444	0.02	0.032	0.485
Fasting time	0.05	0.032	0.148	0.04	0.032	0.251	−0.03	0.033	0.341	−0.03	0.033	0.303	−0.01	0.033	0.816	0.00	0.033	0.974	0.04	0.032	0.26	0.03	0.032	0.399
Waist:hip				0.09	0.034	**0.008**				0.03	0.035	0.458				−0.08	0.035	**0.025**				0.08	0.034	**0.017**
Intercept	0.00	0.035	**<0.001**	0.00	0.035	**<0.001**	−0.01	0.036	**<0.001**	−0.01	0.036	**<0.001**	0.00	0.034	**<0.001**	0.00	0.033	**<0.001**	0.00	0.032	**0.002**	0.00	0.032	0.404
Sex	0.11	0.034	**0.001**	0.11	0.034	**0.001**	−0.02	0.035	0.611	−0.02	0.035	0.593	0.06	0.035	0.111	0.06	0.035	0.098	0.14	0.034	**<0.001**	0.13	0.034	**<0.001**
SEP	−0.07	0.033	**0.028**	−0.06	0.033	0.078	−0.07	0.033	**0.041**	−0.06	0.033	0.06	0.09	0.033	**0.009**	0.07	0.033	**0.026**	−0.15	0.032	**<0.001**	−0.13	0.033	**<0.001**
Age	−0.05	0.033	0.18	−0.04	0.033	0.184	0.05	0.034	0.105	0.06	0.034	0.104	−0.01	0.034	0.677	−0.02	0.033	0.659	0.06	0.033	0.088	0.06	0.033	0.082
Leucine	−0.01	0.034	0.791	−0.03	0.035	0.331	−0.02	0.034	0.65	−0.02	0.035	0.492	−0.02	0.034	0.554	0.00	0.035	0.977	0.09	0.034	**0.011**	0.06	0.035	0.072
Alcohol use	0.06	0.032	0.062	0.06	0.032	0.067	−0.05	0.032	0.095	−0.06	0.032	0.092	0.02	0.033	0.496	0.02	0.032	0.468	0.02	0.032	0.544	0.02	0.032	0.562
Fasting time	0.05	0.032	0.165	0.04	0.032	0.274	−0.03	0.033	0.302	−0.04	0.033	0.258	−0.01	0.033	0.835	0.00	0.033	0.955	0.04	0.032	0.268	0.03	0.032	0.414
Waist:hip				0.09	0.034	**0.006**				0.03	0.034	0.328				−0.08	0.034	**0.019**				0.09	0.034	**0.009**
Intercept	0.00	0.035	**<0.001**	0.00	0.035	**<0.001**	−0.01	0.036	**<0.001**	−0.01	0.036	**<0.001**	0.00	0.034	**<0.001**	0.00	0.033	**<0.001**	0.00	0.032	**0.001**	0.00	0.032	0.419
Sex	0.12	0.034	**0.001**	0.12	0.034	**<0.001**	−0.02	0.034	0.503	−0.02	0.034	0.507	0.06	0.034	0.068	0.06	0.034	0.071	0.13	0.034	**<0.001**	0.13	0.034	**<0.001**
SEP	−0.07	0.033	**0.029**	−0.06	0.033	0.084	−0.07	0.033	**0.042**	−0.06	0.033	0.067	0.09	0.033	**0.008**	0.07	0.033	**0.027**	−0.15	0.032	**<0.001**	−0.14	0.033	**<0.001**
Age	−0.04	0.033	0.183	−0.04	0.033	0.187	0.05	0.034	0.109	0.05	0.033	0.108	−0.01	0.034	0.686	−0.01	0.033	0.667	0.06	0.033	0.089	0.06	0.033	0.082
Valine	0.01	0.033	0.842	−0.01	0.034	0.795	−0.04	0.034	0.251	−0.05	0.034	0.19	0.00	0.034	0.909	0.02	0.034	0.59	0.06	0.033	0.058	0.05	0.034	0.173
Alcohol use	0.06	0.032	0.062	0.06	0.032	0.07	−0.06	0.032	0.088	−0.06	0.032	0.083	0.02	0.033	0.503	0.02	0.032	0.461	0.02	0.032	0.485	0.02	0.032	0.521
Fasting time	0.05	0.032	0.151	0.04	0.032	0.238	−0.04	0.033	0.266	−0.04	0.033	0.226	0.00	0.033	0.891	0.00	0.033	0.905	0.03	0.032	0.289	0.03	0.032	0.444
Waist:hip				0.09	0.033	**0.009**				0.04	0.034	0.295				−0.08	0.034	**0.013**				0.10	0.033	**0.004**

*Note*: Random intercepts used to adjust for clustering by postcode.

Abbreviations: SE, standard error; SEP, socioeconomic position; Waist:hip, waist‐to‐hip ratio; β, standardised coefficient.
